# The Potential of Edible Films, Sheets, and Coatings Based on Fruits and Vegetables in the Context of Sustainable Food Packaging Development

**DOI:** 10.3390/polym15214231

**Published:** 2023-10-26

**Authors:** Monika Janowicz, Sabina Galus, Agnieszka Ciurzyńska, Małgorzata Nowacka

**Affiliations:** Department of Food Engineering and Process Management, Institute of Food Sciences, Warsaw University of Life Sciences—SGGW, 159c Nowoursynowska St., 02-776 Warsaw, Poland; monika_janowicz@sggw.edu.pl (M.J.); agnieszka_ciurzynska@sggw.edu.pl (A.C.)

**Keywords:** edible film, edible sheet, edible coating, fruits, vegetables, food packaging

## Abstract

Several consumable substances, including fruit and vegetable purees, extracts, juices, and plant residue, were analyzed for their matrix-forming potential. These matrices serve as the basis for the production of edible films, sheets, and coatings that can be eaten as nutritional treats or applied to food products, thereby contributing to their overall good quality. Furthermore, this innovative approach also contributes to optimizing the performance of synthetic packaging, ultimately reducing reliance on synthetic polymers in various applications. This article explores the viability of incorporating fruits and vegetables as basic ingredients within edible films, sheets, and coatings. The utilization of fruits and vegetables in this manner becomes achievable due to the existence of polysaccharides and proteins that facilitate the formation of matrices in their makeup. Moreover, including bioactive substances like vitamins and polyphenols can impart attributes akin to active materials, such as antioxidants or antimicrobial agents. Advancing the creation of edible films, sheets, and coatings derived from fruits and vegetables holds great potential for merging the barrier and mechanical attributes of biopolymers with the nutritional and sensory qualities inherent in these natural components. These edible films made from fruits and vegetables could potentially serve as alternatives to seaweed in sushi production or even replace conventional bread, pancakes, tortillas, and lavash in the diet of people suffering from celiac disease or gluten allergy, while fruit and vegetable coatings may be used in fresh and processed food products, especially fruits and vegetables but also sweets.

## 1. Introduction

The quick development of civilization is generally perceived as positive and desirable, and increases the duration and quality of human life [1]. In the last few decades, the global average life expectancy has increased dramatically in all countries. It has risen from about 52 years for people born in 1960 to more than 70 years at present [2]. Nonetheless, the progress of civilization, especially industrialization, urbanization, and nutrition, has also become a new source of adverse effects on human health. Modern science has eliminated the threat of death from most infectious diseases, but it is now struggling with lifestyle diseases such as heart diseases, stroke, and cancer, which are a result of an inappropriate relationship of people with their environment. Fortunately, civilization diseases differ significantly from other diseases since they are considered preventable, and their morbidity rate may be lowered by changes in lifestyle, eating habits, and the environment [3]. Modern lifestyle diseases are a group of diseases that arise from various causes and their occurrence is especially common in developed countries, where the lifestyle is based on comfort and lack of physical activity. Lifestyle diseases are primarily diseases of the circulatory system, such as hypertension, heart disease, and atherosclerosis, but also obesity, type 2 diabetes, respiratory diseases, and many others, affecting both older people and, increasingly, younger generations. Among them, many people ask themselves the question: how to prevent lifestyle diseases? Prevention primarily includes lifestyle changes, a healthy diet rich in vegetables and fruits, the use of vitamins and antioxidants, limiting the consumption of fats and sugars, regular physical activity, and avoiding stimulants such as cigarettes and alcohol; these are the basic principles of preventing lifestyle diseases. Regular examinations are also important to detect diseases early and prevent their development. That is why it is so important to educate society about a healthy lifestyle and introduce preventive measures that will prevent lifestyle diseases and improve the quality of life [3,4,5].

It is estimated that one-third of cancer cases and about half of cardiovascular disease cases are considered to be diet-related [4]. According to the World Health Organization, a change in eating habits, physical activity, and tobacco control may significantly reduce the rates of chronic diseases, which are responsible for 70% of all deaths globally [5]. An abundance of epidemiological research has demonstrated a negative correlation between chronic diseases and the consumption of fruits and vegetables, indicating an increasing trend in global health promotion. However, not only a deep interest in the general health benefits of fruit and vegetable consumption, but also in understanding the type, number, and mode of action of the different components in fruits and vegetables that provide health benefits have been noted [4].

At present, sustainable development stands as a paramount guiding principle for policymakers, manufacturers, researchers, and the general public alike. Nevertheless, as per the statistics from the Food and Agriculture Organization of the United Nations, approximately 1.3 billion tons of food are discarded on a global scale each year [6]. Notably, fruits, vegetables, roots, and tubers exhibit the highest rates of wastage among all food products, with nearly half of the total production of fruits and vegetables going to waste [7]. Within the fruits and vegetables category, losses and wastage manifest across every stage of the food supply chain (FSC). These stages encompass agricultural production, post-harvest handling and storage, processing (resulting in food losses), and distribution and consumption (leading to food waste) [8]. However, their amounts, in general and in particular segments of FSC, differ significantly depending on the region–its industrialization, climate conditions, and society wealth. Industrialized regions, among which Europe, North America, and Oceania, as well as industrialized Asia are mentioned, struggle mainly with losses in agricultural production caused mostly by post-harvest fruit and vegetable grading resulting from quality standards set by retailers. The second substantial problem is fruit and vegetable waste at the end of the FSC, with 15–30% of purchases by mass discarded by consumers. On the other hand, developing countries are mainly dominated by losses in agricultural production, as well as during post-harvest and distribution stages, which is related to financial, managerial, and technical limitations in harvesting techniques, storage, and cooling facilities in difficult climatic conditions, infrastructure, packaging and marketing systems [6].

**Figure 1:**
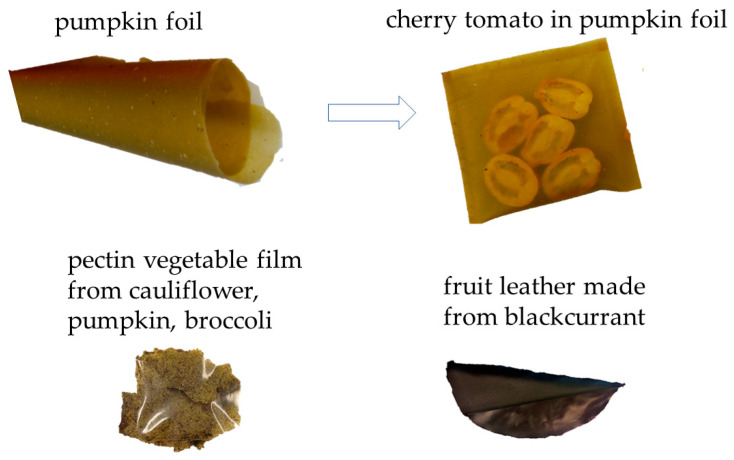
Examples of the fruit and vegetable waste packaging (own data and photographs).

The extent of fruit and vegetable losses, as well as waste, bears significance not just due to the social and economic disparities that result in global poverty and hunger, but also due to their role in climate change through heightened greenhouse gas emissions. This is compounded by excessive production and the wasteful utilization of significant natural resources. Some of the losses are generated during the preparation of fruit and vegetables for consumption, and the waste consists of parts unfit for consumption, the percentage of which depends on the type of raw material. In this way, up to 5% of the initial weight of green beans, 16% of potatoes, 19% of carrots, and even up to 46% of the weight of pineapple and 66% of green peas are lost. This amount varies both in general and at individual stages of the supply chain depending on the region—its degree of industrialization, climatic conditions, and the wealth of society. Industrialized regions, such as Europe, North America, and Oceania, as well as the industrialized part of Asia, also struggle with the losses of raw materials at the stage of agricultural production and post-harvest sorting, which result from the high quality standards set for fruit and vegetables by their commercial recipients (store chains) [9,10,11,12,13]. The above facts are confirmed by the latest FAO Global Food Waste report from 2023, which indicates that over a third of all food produced (~2.5 billion tons) is lost or wasted each year. One third of this occurs in the food production stage. Boston Consulting Group (BCG) estimates this wasted food is worth $230 billion. Researchers estimate the lost food calories from food waste amount to roughly 24% of the total available food calories. To put this in perspective, the UN reports that about a third of the global population, mostly in developing countries and low income countries, did not have enough access to food in 2020–an increase of 320 million people from the previous year, yet wasted food still has enormous processing potential in the context of the sustainable management of raw materials of plant and animal origin [14]. Furthermore, owing to their high biodegradability, fruit and vegetable losses and waste present pressing challenges in terms of disposal and environmental impact, contributing to the accumulation of larger quantities of waste in landfill sites [15]. As a result of this, in recent years, the formulation of strategies and approaches for fruit, vegetable, and side-stream products, as well as waste handling, have emerged as topics of extensive discussion across various administrative tiers, functional domains, and academic fields [8,16,17,18,19]. In the present day, a waste hierarchy comprising six waste management strategies has taken shape: prevention, reduction, reuse, recycling, energy recovery, and disposal [20]. Some of the examples of the fruit and vegetable waste are used to produce packaging (Figure 1). In addition to traditional methods such as donating to those in need [21], extracting specific compounds [22], or producing biogas [23], there is another approach to address the issues related to the overproduction, seasonality, and perishability of fruits and vegetables. This innovative approach is to use fruits and vegetables as primary components for creating edible films, sheets, and coatings. These can serve various purposes, and are not only interesting but also hold promise in the context of sustainable development [24,25].

However, to date, a comprehensive review encompassing the extensive body of research on fruit- and vegetable-based edible films, sheets, and coatings, has not been carried out. In particular, there is no comprehensive review focusing on the unique attributes of fruits and vegetables as sources of the matrix, not taking anatomical and physiological classification, but only classifying the product and the possibility of using fruits and vegetables as structural components and forming bio-active compounds. These edible materials have the potential to enhance the quality and visual appeal of various food products, in addition to prolonging their shelf life. They can also be employed as stand-alone items, serving as sandwich wraps or even as substitutes for conventional pancakes. Furthermore, certain fruit and vegetable forms, such as puree or juice, can be integrated into the matrix-forming solution, offering potential cost and time savings in the production process. Consequently, this study delves into the role played by specific chemical constituents found in fruits and vegetables in shaping the physicochemical and functional characteristics of these edible materials. This study also traces the chronological evolution of these innovative materials and addresses the variations in nomenclature between countries, underscoring the need for standardization in the domain of legal regulations. Additionally, the review provides an analysis of the advantages and disadvantages associated with the use of fruits and vegetables as components within matrix-forming solutions, explores potential applications, outlines future trends, and discusses pivotal considerations for commercialization within the context of sustainable development.

## 2. Fundamentals of Edible Films, Sheets, and Coatings

**Figure 2:**
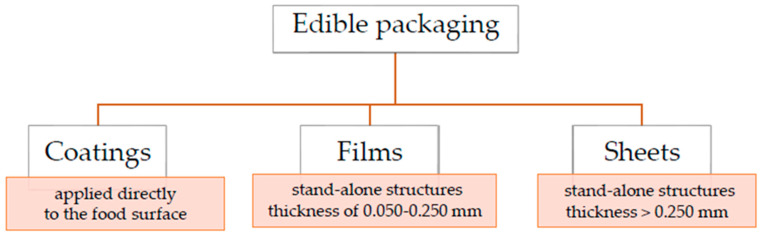
Type of edible packaging produced from biopolymers.

Edible packaging can be described as initial packaging material crafted from biopolymers. A layer of edible substance can be applied directly onto food items or shaped into an independent structure to function as food packaging or a food wrap [18,26,27]. Edible packaging can be categorized into two primary classifications: coatings and films. The distinction between these two categories is based on their application methods to food products. Coatings are used in liquid form and applied to the food product, whereas films are created as separate entities and subsequently applied (Figure 2) [28,29]. Furthermore, certain researchers differentiate edible films based on their thickness. Films are described as stand-alone layers with a thin composition (0.050–0.250 mm), whereas those with greater thickness (>0.250 mm) are referred to as sheets [23,26,30]. The first mention of the use of edible lipid coatings on citrus in China comes from the twelfth century [31]. Nonetheless, the concept of applying edible films/sheets/coatings to food products gained increasing interest among scientists only in the 1950s [32]. This may be due to the fact that in recent decades, humankind has faced issues such as lifestyle diseases and environmental problems that have become the driving force behind much scientific research. Increasing consumer awareness of food and environmental influence on their health has forced scientists and companies to explore different ways to produce natural foods combining environmental benefits and sustainability. One of the more fashionable trends is the use of biopolymers derived from renewable resources in multiple food-packaging applications, among which edible films/sheets/coatings are mentioned [33,34].

There are a great number of benefits achieved by the application of edible coatings, films and sheets to food products. They may be exploited as moisture, gas, and solute barriers extending the shelf-life of various food products [35]. Edible films, sheets, and coating can also function as carriers for food additives, including aromas, colorants, flavors, spices, and nutrients, as well as antimicrobial and anti-browning agents. This enhances the overall quality of food products, elevates sensory experiences, and adds to convenience [36]. Recent trends in edible packaging research have indicated a focus on multi-component or composite materials, which aim to harness the advantages of each component while minimizing their drawbacks [37]. There is also an emphasis on incorporating active materials with antimicrobial properties, particularly essential oils [38], exploring cross-linking techniques [39], and employing nanoreinforcement to produce bionanocomposites [40]. Another noteworthy trend which is linked with sustainable development involves the use of fruits and vegetables in the creation of edible films, sheets, and coatings [41,42,43].

## 3. Confusing Nomenclature

The most frequent way of classifying edible films, sheets, and coatings is according to their structural material. Edible films are differentiated by their proteins, polysaccharides, lipids, and composite structures. The wide interest of companies and scientists in such materials is mainly due to their biodegradability, but also their relatively low cost of production, the abundance of biopolymers in nature, and the high consumer demand for natural and healthy foods [26,34]. However, biopolymers also have many other than uses than as binding agents, such as as antimicrobials [35], plasticizers [44], crosslinkers [45], or particular nutrients [46] incorporated into the polymer matrix of edible films, sheets, and coatings.

Fruits and vegetables possess the potential to act as multifunctional additives in edible films. The initial scientific exploration of fruit- and vegetable-based edible packaging materials, referred to as films or coatings, emerged in the 1990s [47]. Over the past two decades, numerous studies on diverse edible materials derived from fruits and vegetables have been documented (Table 1). Nevertheless, it is reasonable to assume that structures resembling films or sheets, such as fruit leather and pestil, have been in existence for a much more extended period. Notably, research into the production of fruit leathers can be traced back to the 1970s [48]. Since then, their physicochemical properties, sensory attributes, quality, and stability during storage, as well as combined preservation technologies, have been the object of many studies [49,50,51,52,53,54]. Generally, fruit leathers are dehydrated fruit-based products obtained by drying a thin layer of fruit puree until a leathery consistency is obtained [55]. The attractive texture of soft leather is achieved by the acid-sugar-high methoxyl pectin gelation [56]. Fruit leathers mainly consist of carbohydrates such as sugars, pectins, and cellulosic substances [55]. The production of fruit leathers is considered one of the oldest methods used to prolong the shelf life of fruits thanks to their intermediate water activity, low moisture content, and low pH, guaranteeing microbial stability [57,58]. Nowadays, fruit leathers are widely consumed as candies or snacks in many parts of the world, fortifying the daily diet with dietary fiber, vitamins, and minerals, as well as providing a substantial energy input [58,59,60]. Recently, the popularity of fruit leathers has increased, transforming them from a homemade product into an industrial one and modifying their original formulation by adding new ingredients such as corn syrup, honey, citric pectin, maltodextrin, lecithin, vegetable oils, and ascorbic acid, among others [58,60].

Pestil is a traditional Turkish dried fruit snack produced from different types of fruit, e.g., mulberry, plum, apple, and apricot. However, it is grape pestil that is the most important. Although some authors use the names fruit leather and pestil interchangeably [57,61] there are some differences between them. According to the Food and Agriculture Organization of the United Nations, fruit leathers are dried sheets of fruit pulp/puree that have a soft, rubbery texture and a sweet taste [62]. A variety of ingredients can be added to the fruit puree, such as sugar (which increases the sweetness), citric acid (which increases the acidity), and chopped nuts, coconut, and spices (which vary the taste and flavor). On the other hand Kaya, Fahrettin, and Madeni [63] described two products based on fruit juices, pestil and foamed pestil. They are both prepared from fruit juice concentrate and then mixed with starch or wheat flour, respectively. The foamed pestil is obtained by whipping the mixture of juice and wheat starch while cooking, which provides a white color. This type of pestil can be also produced with the addition of nuts (1 ± 2% of overall weight). What is more, pestil may be also used as a coating for nuts, and such a product is known as sucuk [64]. On the other hand, Diamante, Bai, and Busch [48], in their review article summarizing published information about fruit leathers, define pestil as a synonym for grape leather, with no mention of pestil based on other fruits. What is more, a host of innovative terms has emerged to describe edible packaging materials. Chinese scientists commonly refer to these materials as “vegetable paper,” describing compositions that incorporate binding agents, plasticizers, and vegetable pastes or purees [65,66,67]. In the production of edible wrapping and packaging paper, ingredients such as celery, Chinese chive, and Dangshan pear have been utilized [68,69,70]. In addition to these, less commonly used expressions such as “dehydrated matrix” [71] or simply plastic [72] have also been introduced to represent similar ideas in this domain.

The wide variety of nomenclature used to describe structures derived from fruits and vegetables, either in combination with or without binding agents, plasticizers, or other additives, can create confusion when attempting to categorize a particular product. This is particularly challenging in regions where products like fruit leather or pestil are unfamiliar, like Poland. However, it is essential to note that, primarily, edible films, coatings, and papers are typically portrayed as products designed to enhance food quality [73] and offer an environmentally friendly packaging alternative [70]. In contrast, fruit leathers, pestil, and sucuk are generally recognized as nutritious snacks or confectioneries [55,64].

To summarize, it may be stated that edible films, coatings and papers are rather more advanced forms of fruit leathers and pestils thanks to far more complex formulations and production techniques, as well as their later development. Otoni, Avena-Bustillos, Azeredo, Lorevice, Moura, Mattoso, and McHugh [74] proposed the application of fruit and vegetable edible films as leathers, which seems to be synonymous with snacks/candies. However, it appears to be worth considering fruit and vegetable leathers as sheets, and changing their role from sweet snacks into nutritious food wraps for sandwiches or alternatives for pancakes or tortillas on a gluten-free diet. This approach is worth further exploration in future studies, also because there is much more research on films and coatings rather than sheets. The authors would also like to emphasize the necessity of clear classification of described structures as well as legal regulations, especially in the European Union where there is no official definition of edible film, paper, or packaging.

**Table 1:** Fruit and vegetables as a compound of edible packaging.

Common Name	Form of Fruit/Vegetable Used	Form of the Final Product	References
Açaí	puree	film	[75,76]
Acerola (Barbados cherry)	puree	film	[77,78,79,80]
		film/coating	[81]
		film/heat-sealed sachet	[82]
	alcoholic extract from whole fruit	film	[83]
	flour (freeze-dried powdered whole fruit)		[84]
Apple	puree	film	[85,86,87,88,89,90]
		coating	[88,91]
		film/film wrap/coating	[73]
		film/film wrap	[92,93]
		leather	[55,56,58,60,71,94]
	pomace (convective dried powdered peel)	film	[95]
		coating	[43]
Apricot	puree	film	[47]
	clarified juice	pestil/leather/sheet	[59]
		pestil/leather	[61]
Banana	puree	film	[96]
			[97]
			[98]
	flour (convective dried powdered fruit without peel)	film	[41,99,100,101]
		film heat-sealed sachet	[102,103,104]
Broccoli	puree	film	[90]
Cabbage	^NA^	vegetable paper	[65]
Carrot	pomace (convective dried powdered solid residue generated by the processing of whole vegetables during juice production)	film	[105]
	puree	film/film wrap	[93]
		film	[89,90,106]
		coating	[107]
		carrot paper	[108]
	pomace (convective dried powdered solid residue derived from minimally processed carrots)	film	[109]
	pomace (convective dried powdered solid residue generated by the processing of whole vegetables during juice production)	film/coating	[110]
		film/film packaging/coating	[111]
Cashew apple	alcoholic extract from whole fruit	film	[83]
Celery	puree	wrapping paper	[68]
Chinese chive	puree	packaging paper	[70]
Courgette	pomace (convective dried powdered solid residue generated by the processing of whole vegetables during juice production)	film	[105]
		film/film packaging/coating	[111]
		film/coating	[110]
Cranberry	pomace extract	film	[112]
Cucumber	pomace (convective dried powdered solid residue generated by the processing of whole vegetables during juice production)	film	[105]
		film/film packaging/coating	[111]
		film/coating	[110]
Durian fruit	puree	leather	[113]
Fennel	homogenized residue	film	[114]
Grapes	pomace extract	film	[115,116]
	clarified juice	pestil/leather/sheet	[59]
		pestil/leather	[61,63,64,117,118]
Guava	puree	film	[119]
Hibiscus	puree	film	[89]
		film/film wrap	[93]
Indian gooseberry	puree/extract	film/coating	[120]
Jackfruit	^ND^	leather	[121]
	puree		[122]
Jocote	puree	film/film packaging	[78]
Kiwi	puree	leather	[123,124]
Lettuce	pomace (convective dried powdered solid residue generated by the processing of whole vegetables during juice production)	film	[105]
		film/film packaging/coating	[111]
		film/coating	[110]
	residue	paper	[67]
Longan	puree	leather	[54]
Mango	puree	leather	[125]
		film	[78,126]
		film/film wrap	[127,128]
		film/heat-sealed sachet	[82,129]
Mint	pomace (convective dried powdered solid residue generated by the processing of whole vegetables during juice production)	film	[105]
		film/film packaging/coating	[111]
		film/coating	[110]
Mulberry	clarified juice	pestil/leather/sheet	[59]
		pestil/leather	[61]
Orange	pomace (convective dried powdered solid residue generated by the processing of whole vegetables during juice production)	film	[105]
		film/film packaging/coating	[111]
		film/coating	[110]
Papaya	puree	leather	[130]
		film	[131]
		heat-sealed sachet	[132]
Passion fruit	pomace (convective dried powdered solid residue generated by the processing of whole vegetables during juice production)	film	[105]
		film/film packaging/coating	[111]
		film/coating	[110]
Peach	puree	film	[47,90]
Pear	juice concentrate	leather	[133]
	puree	film	[47]
		wrapping paper	[69]
Pineapple	puree	leather	[134,135]
Plum	clarified juice	pestil/leather	[61]
Pomegranate	juice	film	[136]
	juice concentrate	leather/pestil	[137,138]
Potato	pomace (convective dried powdered peel)	film/film packaging/coating	[111]
Pumpkin	residue extract	film/film packaging	[139]
	pomace (convective dried powdered solid residue generated by the processing of whole vegetables during juice production)	film/coating	[110]
	^NA^	paper	[66]
Quince	puree	leather	[58]
Rocket	pomace (convective dried powdered solid residue generated by the processing of whole vegetables during juice production)	film	[105]
		film/film packaging/coating	[111]
		film/coating	[110]
Rosehip	puree	leather	[140,141]
Spinach	pomace (convective dried powdered solid residue generated by the processing of whole vegetables during juice production)	film	[142]
		film/film packaging/coating	[111]
		film/coating	[110]
Strawberry	puree	leather	[123,143]
	alcoholic extract from whole fruit	film	[83]
Sugar beetroot	puree	film	[144]
	residue extract		[145]
	residue		[72]
Taro	pomace (convective dried powdered solid residue generated by the processing of whole vegetables during juice production)	film	[105]
		film/film packaging/coating	[111]
		film/coating	[110]
Tomato	puree	film	[146,147]
		coating	[91]
Watermelon	pomace (convective dried powdered solid residue generated by the processing of whole vegetables during juice production)	film	[105]
		film/film packaging/coating	[111]
		film/coating	[110]

^NA^–not available, ^ND^–not disclosed.

## 4. Fruits and Vegetables as a Source of Functional Compounds of Edible Materials

Edible films, sheets, and coatings should be composed of at least two components: a binding agent that enables the formation of a cohesive structure (mainly protein and polysaccharide hydrocolloids) and a solvent (usually water) [26,148]. A plasticizer that reduces the brittleness inherent to most biopolymers is often essential [44]. There is also a great number of possible additives modifying particular properties of these materials [24,74].

**Figure 3:**
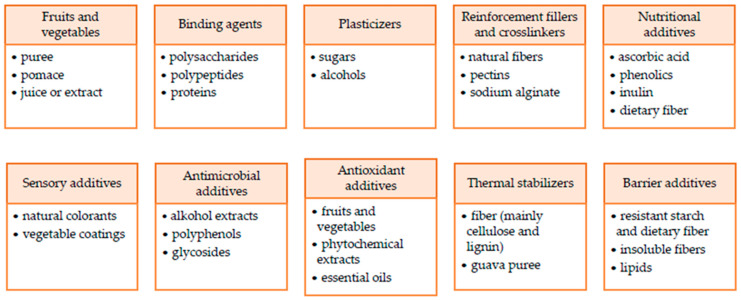
Components used for creating edible materials and examples of these materials.

Thanks to their rich chemical compositions, fruits and vegetables may serve as an interesting source of edible film compounds of any kind [126]. In Table 2, the chemical composition of some fruits and vegetables is presented to assess their potential to serve as a multifunctional component of edible materials. However, not only the presence of particular compounds but also the interactions between them resulting from mutual ratio are important [103]. Complex characteristics of particular chemical compounds that can be found in plant tissue are presented below to facilitate predicting and designing the properties of edible films and coatings based on fruit and vegetables. Also, the components used for creating edible materials are presented in Figure 3.

### 4.1. Binding Agents

Numerous components found in fruits and vegetables, including pectin, cellulosic substances, starch, and proteins, possess the capability to create cost-effective, renewable, biodegradable edible materials that serve as effective barriers against oxygen [103]. The plant cell wall comprises pectins, esteemed as among the most intricate macromolecules, that enhance tissue integrity and stiffness. Notably, apple pomace and citrus peel stand as prominent industrial sources of this particular macromolecular component [76]. The apple parenchyma is rich in pectin, with concentrations ranging from 0.15 to 0.25 kg/kg of dry matter. Quince is likewise a substantial source of this compound, exceeding 0.15 kg/kg of dry matter. In contrast, bananas contain a lower quantity of pectin, which further diminishes during ripening, declining from approximately 12 to 6.7 mg per gram of fruit. Pectin plays a vital role in the formulation of the matrix within edible materials, ultimately resulting in what can be described as partially dehydrated pectic gels [58,60,96]. For example, sugar beet pulp, derived from the sugar extraction process, emerged as an exceptional ingredient for the biopolymer matrix due to its abundant cell wall polysaccharides, including arabinoxylans hemicelluloses and highly methylated and acetylated pectin, utilized in the fabrication of extruded film strips [144]. In unripe bananas, starch constitutes the primary constituent, comprising over 70% of the dry matter. However, as the fruit ripens, this starch undergoes conversion into soluble sugars, thereby influencing the plasticizing influence within the fruit component of the matrix-forming solution. Additionally, the ratio of amylose to amylopectin within the overall starch mass is noteworthy, as it is primarily amylose that plays a pivotal role in determining the matrix-forming capacity of starches.

Edible films, sheets, and coatings, when infused with a natural blend of binding agents, lipids, and bioactive compounds from fruits and vegetables, inherently become composite structures. This inherent composite nature holds the potential to address the issues of film brittleness and phase separation that often arise in synthetically formulated biopolymer solutions. These problems stem from the thermodynamic incompatibility between lipids and proteins or polysaccharides in artificial designs [100,104]. As an illustration, within a cellulosic environment, pectin could establish intermolecular connections between its homogalacturonan segments and the cellulose chains and microfibrils. Additionally, pectin can offset any adverse effects caused by the presence of plasticizers, such as glycerol [96]. For example, pectin and proteins, naturally present in bananas, have the potential to create crosslinks with each other, thereby enhancing the mechanical properties of edible films, sheets, and coatings [103]. Pectin and starch chains can also have a beneficial impact on the mechanical properties of edible films by forming hydrogen bonds. When a higher content of starch is used in the matrix solution, the resulting material exhibits increased mechanical resistance due to enhanced cohesiveness [154]. Pelissari, Andrare-Mahecha, do Amaral Sobral, and Menegalli [101] conducted an assessment of banana flour films as laminated materials in comparison to banana starch films. The non-uniform, more porous, and open structure resulting from interactions between specific components (e.g., starch protein, starch cellulose/fiber, amylose lipids) can exert varying influences on the optical, mechanical, and barrier properties, as well as sealability, of the final structure. However, Otoni, Avena-Bustillos, Azeredo, Lorevice, Moura, Mattoso, and McHugh [74] emphasized that materials composed solely of fruit/vegetable puree typically exhibit inadequate barrier properties, lack consistency, and possess limited mechanical strength, rendering them unsuitable for various applications. Consequently, edible materials based on fruits and vegetables often necessitate the addition of binding agents to enhance film cohesiveness [77,102,106,146].

### 4.2. Plasticizers

Water molecules are naturally present in all fruits and vegetables, and due to their low molecular weight, they function as natural plasticizers. This characteristic increases the free volume, providing greater mobility and flexibility to the macromolecules within the biopolymer matrix. Consequently, the glass transition temperature (Tg) of the material decreases as the water activity rises, resulting in decreased rigidity. However, a phenomenon known as “moisture toughening” can also occur when hydration facilitates a molecular rearrangement, leading to interactions between water and the matrix macromolecules. This effect has been observed in products like edible films, sheets, and coatings, such as apple leather strips, at relative humidity levels between 11 and 33% [55,101].

In addition to water, fruits contain other natural plasticizers, including low-molecular-weight sugars such as glucose, fructose, and sucrose. These sugars can lower the Tg and create sticky points in the material. Low Tg values are desirable for enhancing the flexibility of fruit-based edible films, especially when the product needs to be handled or folded, as is the case with food wraps used at both ambient and refrigeration temperatures [94]. The presence of plasticizing sugars from sources like papaya puree and pomegranate juice has been found to decrease the values of elastic modulus and tensile strength while increasing the values of elongation at break in pectin films [131,136]. Beyond sugars, proteins and lipids found in certain fruits and vegetables, such as bananas, can also act as plasticizers, impacting the overall performance of the final film, sheet, or coating [154,155]. Furthermore, the addition of grape pomace extract to starch edible films has been shown to significantly increase film flexibility, with phenolic compounds from the extract also suspected to serve as plasticizers [116].

### 4.3. Reinforcement Fillers and Crosslinkers

For reinforcement fillers and crosslinks, different natural fibers, pectins, sodium alginate, and other materials can be used. For example, isolated açaí seed fibers have been utilized as strengthening additives in the production of recycled thermoplastics like high-impact polystyrene (HIPS) cups and polypropylene (PP) bottles. When these natural fibers, rich in lignin and hemicelluloses, were incorporated into the polymer matrix, it led to reduced tensile strength and compression resistance compared to recycled polymers without fibers. This was attributed to an inadequate bond between the fibrous layer and the recycled material, resulting in delamination and fiber displacement. However, the presence of açaí seed fibers in recycled thermoplastic formulations demonstrated a notable increase in impact resistance, with PP experiencing a 44% improvement and HIPS a 12% enhancement [156]. In contrast, research by Espitia, Avena-Bustillos, Du, Teófilo, Soares, and McHugh [76] showed that incorporating apple skin powder, a rich source of dietary fiber consisting mainly of cellulose, hemicelluloses, lignins, pectins, and gums, into açaí pectin edible film improved its tensile strength. Conversely, Iahnke, Costa, Rios, and Flôres [109] found contrasting results when they added powdered carrot residue, which is abundant in dietary fiber (75 g/100 g of dry matter), to gelatin films. They observed a positive linear correlation between the vegetable compound and the Young Modulus, indicating increased stiffness in the film. However, this simultaneous effect led to reduced elongation at break and tensile strength values, resulting in a less flexible and weaker structure. This unusual behavior was attributed to the patchy morphology of the films with carrot residue powder, which hindered the natural fiber’s ability to serve as a reinforcing filler [157].

Fruit and vegetable by-products comprise natural combinations of polysaccharides and proteins, which contribute to additional hydrogen bonding interactions among the polymer chains, thereby enhancing the overall strength of the films [103,158]. The discrepancies observed in the aforementioned results may arise from variations in polarity among the constituents within the composite materials [159]. Tulamandi, Rangarajan, Rizvi, Singhal, Chattopadhyay, and Saha [132] put forward a hypothesis suggesting that the amalgamation of diverse carbohydrates, proteins, and other organic substances in composite papaya films fosters superior cross-linking compared to the mono- or bi-components found in biodegradable films. Further research is required to gain a deeper understanding of the interactions among specific components of the edible matrix and the mechanisms involved in structuring dried gels. This understanding will enable us to predict the ultimate properties of the resulting materials more accurately.

**Figure 4:**
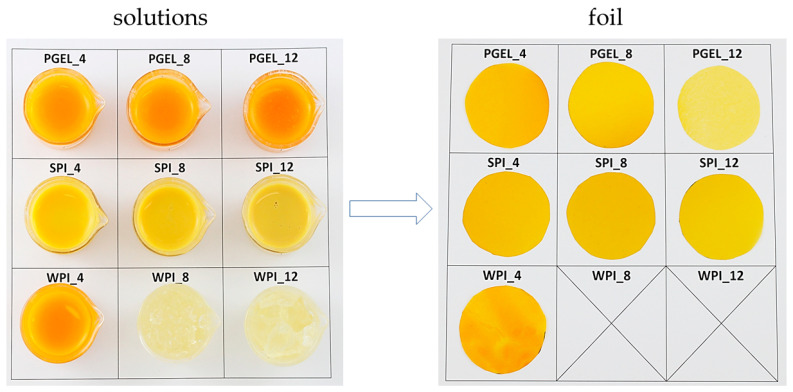
Solutions and dried coatings made from different concentration of gelatin (PGEL_x, where x is 4, 8, and 12%), soy protein isolate (SPI_x, where x is 4, 8, and 12%), and whey protein isolate (WPI_x, where x is 4, 8 and 12%) (own data and photographs).

Certain compounds found in fruits and vegetables are believed to act as crosslinkers. For instance, low methoxyl pectin is recognized for its dependency on multivalent cations to create a gel-like structure. In a study by Park and Zhao [112], they devised films using low-methoxyl pectin combined with cranberry pomace extracts, which served as a source of natural crosslinking agents (including proteins and minerals). The added pectin interacted with components from the pomace extract, resulting in the formation of independent films without the need for additional cationic additives. Similarly, Deng and Zhao [115] developed films using wine grape (cv. Merlot) pomace extract and various binding agents like low-methoxyl pectin, sodium alginate, and Ticafilm^®^ (a blend of sodium alginate, carrageenan, and cellulose gum). Alginates, as derivatives of components of cell walls, and the intracellular matrix of brown seaweed, consisting of ß-d-mannuronic acid and α-l-guluronic acid, are characterized by the ability to act synergistically with substances of other origins, and their properties depend on their sequence of monomers, which makes them a universal material and means that they can be used in the creation of coatings as a result of their interaction with di- and trivalent cations or as agents supporting the swelling in the water of composite materials in the form of films and coatings [160,161]. Although sodium alginate and low-methoxyl pectin typically rely on divalent cations to establish a gel-like structure, the presence of natural crosslinkers in wine grape pomace extract, including minerals, proteins, organic acids, and phenolic acids, obviated the necessity for supplementary crosslinking agents [109]. Figure 4 presents solutions and dried coatings made from different concentrations of gelatin, soy, and whey protein isolate, which determine the specific structure of the final coatings.

### 4.4. Nutritional Additives

Açaí berries have gained popularity thanks to their high content of anthocyanins, which demonstrate antioxidant and anti-inflammatory activity [156]. Acerola is rich in ascorbic acid—a well-known natural antioxidant [77]. It also contains a large number of phenolic compounds and carotenoids, making it the most nutritionally attractive fruit of the fruits (acerola, cashew apple, papaya, pequi, and strawberry) investigated by Eça, Machado, Hubinger, and Menegalli [83]. Du, Avena-Bustillos, Woods, Breksa, McHugh, Friedman, Levin, and Mandrell [91] emphasized that phenolic compounds from apples may prevent cardiovascular disease and cancer, while the consumption of tomatoes and tomato products, as well as isolated bioactive tomato compounds are reported to be related to a lower risk of heart disease, cancer, diabetes, and hypertension. Lorevice, de Moura, Aouada, and Mattoso [119] chose guava as a fruit component of hydroxypropyl methylcellulose films, highlighting five-times-higher content of vitamin C in guava than in the orange, as well as a high content of iron and potassium.

González-Herrera, Rutiaga-Quiñones, Aguilar, Ochoa-Martínez, Contreras-Esquivel, López, and Rodríguez-Herrera [71] indicated that fruit leathers are considered healthy snacks with widely acceptable sensory attributes. What is more, they may serve as a carrier for pre- and probiotics, thus enhancing their beneficial impact on health and increasing their popularity. The authors added agave fructans, inulin, oligofructose, and other mixtures to apple leather. Demarchi, Quintero Ruiz, and Giner [140] drew attention to the possibility of developing the market of simultaneously healthy, nutritional, and comfortable products (fast food/snacks) by designing various fruit types of leather, which enhance the intake of dietary fiber, vitamins, and minerals, add variety to the diet, and provide a substantial energy input. They studied rosehip leathers as a rich source of minerals, anthocyanins, carotenoids, and vitamin C. Summarizing the above, fruits and vegetables are excellent sources of nutrients such as vitamins, trace elements, digestible and indigestible dietary fiber, antioxidative compounds, and sugars, which makes edible materials not only environmentally friendly but also human-friendly [162].

### 4.5. Sensory Additives

Certain fruits and vegetables contain natural colorants, like the anthocyanins found in açaí, which give rise to a spectrum of appealing colors including blue and red [75]. However, these natural colorants, such as anthocyanins, are susceptible to degradation during processing and storage, causing the colors in the final products to fade. To address this issue, Azeredo, Miranda, Ribeiro, Rosa, and Nascimento [81] have suggested the use of montmorillonite as an effective stabilizer for anthocyanins. This stabilization is achieved through electrostatic interactions between flavylium cations and clay platelets, which provide steric protection against degradation reactions. On the other hand, transparency is a desired feature in food packaging, as some consumers prefer to see the contents inside [132]. However, factors beyond colorants, like the presence of amylose, lipids, and dietary fiber, can significantly reduce the material’s transparency [101,109].

**Figure 5:**
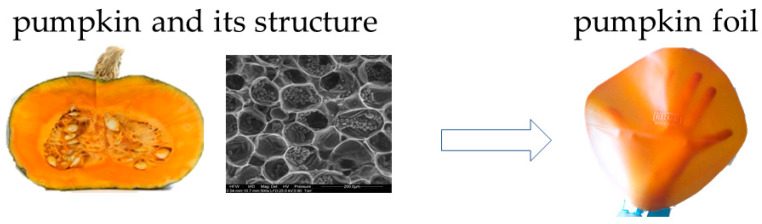
Natural color of pumpkin foil (own data and photographs).

Numerous authors highlight the inherent flavor attributes of apples and tomatoes [91] as distinct advantages of fruit- and vegetable-based edible films. One of the example of the color foil from pumpkin is shown in Figure 5. Furthermore, the taste profiles of diverse food items can also be intentionally crafted through the utilization of fruit and vegetable coatings. Cooked beef patties coated with carboxymethylcellulose-apple peel powder-tartaric acid coatings gained a higher taste score than the control sample, which the authors explained by the apple flavor present in the coating solution formulation [43]. Apple skin polyphenols (ASPs) are one of the components responsible for the flavor of apples. However, the incorporation of high concentrations of ASPs may hurt the palatability of stand-alone films and coated food products by inducing astringency and bitterness [87]. What is more, plant extracts include proteases such as papain, bromelin, ficin, and actinidin, which cause the tenderization of meat. Apple peel powder acted as a proteolytic enzyme, breaking down the myofibrillar proteins of the beef patties and leading to enhanced texture, taste, and overall acceptability in comparison to uncoated samples [43]. Apart from color and flavor, aroma contributes greatly to the product’s attractiveness. Lorevice, de Moura, Aouada, and Mattoso [119] noticed that guava puree films, even after being stored for 10 months, were still characterized by a pleasant guava aroma.

### 4.6. Antimicrobial Additives

Fruits and vegetables can serve as a natural source of antimicrobial compounds, particularly polyphenols, which are categorized as phenolic acid derivatives, flavonoids, or tannins, based on their structure. These polyphenols naturally occur in apple pulp, including catechin, procyanidin, caffeic acid, and chlorogenic acid, as well as in apple skin, which contains the aforementioned compounds along with quercetin glycosides and cyanidin glycosides [156]. Studies by Espitia, Avena-Bustillos, Du, Teófilo, Soares, and McHugh [76] and Espitia, Avena-Bustillos, Du, Chiou, Williams, Wood, McHugh, and Soares [75] have examined the antimicrobial activity of apple skin powder (ASP), which contains over 80% polyphenols. While the individual antimicrobial activity of ASP against *Listeria monocytogenes* is weaker than thyme essential oil, both antimicrobials are necessary to create mechanically robust films with high antimicrobial efficacy. However, it appears that the concentration of polyphenols in preserves like purees is too low to exhibit inhibitory activity against pathogenic bacteria such as *Escherichia coli* O157:H7, *Salmonella enterica*, and *Listeria monocytogenes* [86,92]. Additional research is required to determine the minimum concentration of polyphenols in apple puree necessary to achieve a significant antimicrobial effect, considering that the contribution of apple puree to the film-forming solution is relatively small (26% w/w) [86] and losses occur during puree and edible film production. Similar observations were made by Otoni, de Moura, Aouada, Camilloto, Cruz, Lorevice, Soares, and Mattoso [131] for papaya puree pectin films (with only 3% fruit puree) and for tomato puree pectin films with 30% vegetable paste [147]. Furthermore, the choice of fruit or vegetable used as the raw material for edible films, sheets, and coatings incorporated with additional antimicrobials, such as essential oils, impacts their final antimicrobial activity. Interestingly, apple films exhibited better antimicrobial activity against *Listeria monocytogenes* on ham and bologna than hibiscus films, despite the expectation of strong antimicrobial effects from hibiscus extracts, which were effective against foodborne pathogens in laboratory media. In contrast, carrot films showed the lowest antimicrobial activity [93], potentially because carrot paste did not display any antimicrobial activity against *Escherichia coli* O157:H7 in laboratory buffer or when mixed with ground beef [163]. This suggests that the kind of fruits and vegetables integrated into the matrix-forming solution significantly influences the characteristics of the end product. However, it is crucial to highlight that fruits and vegetables also contain natural compounds that may serve as nutrients for microorganisms, potentially promoting bacterial growth [96,110].

### 4.7. Antioxidant Additives

Fruits and vegetables are renowned as the most significant natural sources of antioxidants, containing a wealth of phytochemical compounds like vitamins and secondary metabolites, such as phenolic compounds, sterols, carotenoids, saponins, and glucosinolates. These compounds are recognized for their capacity to scavenge free radicals. When these fruits and vegetables are properly processed and integrated into the polymer matrix (as opposed to isolating specific compounds), they offer the advantage of synergism, which amplifies the antioxidant potential of individual components. Studies have even suggested a potential synergistic relationship between phenolic compounds and carotenoids [164]. Furthermore, certain compounds like carotenoids, ascorbic acid, and phenolic compounds may have the ability to regenerate one another due to distinct reaction mechanisms. Additionally, the antioxidants naturally occurring in fruits and vegetables can absorb ultraviolet radiation, thus reducing photooxidation and enhancing the light-blocking properties of edible films and coatings [83]. For instance, Suppakul, Boonlert, Buaphet, Sonkaew, and Luckanatinvong [114] demonstrated that a coating composed of Indian gooseberry extract and methylcellulose was highly effective in extending the shelf-life of roasted cashew nuts by 90 days, thanks to the antioxidant properties of Indian gooseberry, particularly its phenolic functional groups. Gurusiddaiah, Nayaka, Dharmesh, and Salimath [165] identified gallic acid and tannic acid as major antioxidant compounds in Indian gooseberry extract’s phenolic fraction. In another study, Iahnke, Costa, Rios, and Flôres [109] investigated the potential of residues from minimally processed carrots and gelatin capsules to create packaging films. They observed a linear relationship between carrot residue powder and an increase in DPPH radical scavenging activity (from 6.3% to 52.3%), which was attributed to the concurrent rise in carotenoid content, which is known for its natural antioxidant properties. The incorporation of oregano essential oil and pumpkin residue alcohol extract into cassava starch films also significantly enhanced free radical scavenging capacity (from 13.0 ± 0.6% to 58.4 ± 0.3%) [139]. However, in the case of gelatin films enriched with green tea extracts and essential oils, which involve more substantial capital and time investment compared to powdered or pureed fruits/vegetables [166,167], the effect on antioxidant activity was similar. Polyphenol compounds containing electron-repelling groups found in apple peel powder were believed to inhibit lipid oxidation by reducing lipid radicals and ferric ions, and trapping active oxygen species like O_2_ or hydroxyl radicals [43]. Carotenoids in carrot residue powder, when integrated into gelatin films, acted as antioxidants, safeguarding sunflower oil from primary oxidation. The peroxide values remained below 10 mEq/kg (5.32 ± 0.22 mEq/kg after 28 days), which is within the recommended limit for considering refined oils t be fresh [109]. In contrast, despite their in vitro antioxidant activity, bioactive compounds such as oregano essential oil and pumpkin residue alcohol extract added to cassava starch films did not provide sufficient protection against lipid oxidation in ground meat [139].

### 4.8. Thermal Stabilizers

Understanding the thermal properties of edible films, sheets, and coatings derived from fruits and vegetables has significant importance. It not only helps determine their suitability for industrial-scale production and potential applications, but also defines their processability range [74]. In the case of açaí pectin edible films, the fiber content, primarily composed of cellulose and lignin, derived from açaí pulp (12%) and apple skin extract used in the formulation, had a positive impact on thermal stability. This fiber component contributed to the final decomposition stage of açaí pectin edible film degradation, occurring at 400 °C [76]. In contrast, the higher concentration of grape pomace extract had a detrimental effect on the thermal stability of starch films. The grape pomace extract acted as a plasticizer, weakening intermolecular interactions and resulting in a decrease in thermal decomposition temperature (from 291.2 °C for control starch films to 279.7 °C and 284.7 °C for films with an 8% addition of grape pomace extract) [116].

Furthermore, when guava puree was incorporated into hydroxypropyl methylcellulose films, it led to an increase in the glass transition temperature (from 169 °C for pure hydroxypropyl methylcellulose films to 189 °C for films with guava puree), thereby enhancing their thermal stability [119]. However, in the case of papaya starch films, the glass transition temperature was notably lower at 90.19 ± 0.54 °C, and it even decreased when gelatin and defatted soy protein were added [132]. Furthermore, Azeredo, Mattoso, Wood, Williams, Avena-Bustillos, and McHugh [126] reported even lower values, some below freezing, for the glass transition temperature in mango puree films incorporating cellulose nanofibers. This suggests reduced chemical stability due to the high molecular mobility and increased reactivity of the film’s components. Nevertheless, these low values of the glass transition temperature imply the excellent flexibility of the films, even at refrigeration temperatures, which can be advantageous for potential applications as food packaging materials.

### 4.9. Barrier Additives

Carotenoids, anthocyanins, phenolic compounds, and vitamin C, in addition to their antioxidant and nutritional benefits, can also enhance the organization of the polymer matrix by reducing its free volume. This reduction in free volume impedes the passage of water vapor through the film or coating. However, the extent of this effect varies depending on the type of raw material used. For example, pectin films incorporated with cranberry pomace extract showed water vapor permeability values approximately 10 times higher than those with acerola, strawberry, cashew, and apple extracts [83]. Additionally, the hydrophobic nature of certain compounds, such as carotenoids, can significantly lower water solubility and water vapor permeability, as seen in gelatin films incorporated with carrot residue powder [109]. Conversely, Caetano, Hessel, Tondo, Flôres, and Cladera-Olivera [139], who studied active cassava starch films incorporated with oregano essential oil and pumpkin residue alcohol extract rich in carotenoids, observed higher solubility and water vapor permeability. They attributed this to the more porous microstructure of the active films. Orsuwan, Shankar, Wang, Sothornvit, and Rhim [41] also suggested that resistant starch and dietary fiber present in green-stage bananas could increase the hydrophobicity of polymers. In contrast, Reis, de Souza, da Silva, Martins, Nunes, and Druzian [129] found that water vapor permeability decreased in mango puree–cassava starch films due to the interaction between mango pulp fibers and starch, reducing free space within the polymeric matrix and obstructing water vapor passage. However, non-starchy components, especially proteins, were reported to increase the material’s hygroscopic nature [104]. Still, Pelissari, Andrare-Mahecha, do Amaral Sobral, and Menegalli [101] demonstrated that the even higher levels of fiber and protein in banana flour films compared to banana starch films were insufficient to cause greater water vapor diffusion through the matrix. Nevertheless, it is worth noting that the addition of fruits and vegetables to the matrix-forming solution can increase the mobility between polymer chains in the dried matrix due to the presence of sugars acting as plasticizers, potentially leading to increased water vapor permeability [119,131,136].

Fruit and vegetable purees are considered highly polar, making them relatively effective barriers against oxygen but less effective barriers against water vapor. However, Rojas-Graü Raybaudi-Massilia, Soliva-Fortuny, Avena-Bustillos, McHugh, and Martín-Belloso [88] demonstrated that apple puree–alginate edible coatings without essential oils were not effective in reducing the rate of CO_2_ production and O_2_ depletion compared to uncoated samples. These coatings also had a modest inhibitory effect on ethylene production. In contrast, the inclusion of essential oils resulted in a consistently low level of ethylene production.

Another advantageous property of edible packaging is its ability to act as a barrier against light and UV radiation. Amylose molecules tend to form strong hydrogen bonds between the hydroxyl groups of adjacent chains, reducing interactions between the biopolymer and water, and resulting in a non-transparent polymer matrix. Additionally, the presence and concentration of components like lipids and dietary fiber can contribute significantly to the material’s opacity. Opaque materials can serve as effective barriers against incident light, especially for products sensitive to degradation reactions catalyzed by light [101,109].

## 5. Doubts and Questions—Tips for Further Commercialization

Even though the studies on edible packaging have been conducted for quite a long time, more than 20 years, questions and uncertainties still arise at different stages of edible film, sheet, and coating production, including formulation preparation, solution degassing, drying, storage, and application. An emerging trend involves the integration of various functional additives, such as nanoparticles or antimicrobials, into the polymer matrix of edible films and coatings based on fruits and vegetables [75,126,147]. The scientists primarily concentrate on the impact of such additives; however, a few also take into account the influence of specific constituents found in fruits and vegetables. Nonetheless, there is still limited comprehensive information regarding the chemical composition of fruit and vegetable semi-products. Such data would greatly contribute to a more comprehensive understanding of the observed phenomena. The proximate compositions of edible films, sheets, and coatings (depending on the research some of these parameters have been determined: moisture, soluble solids, starch, protein, fat, ash, crude fiber, amylose, carbohydrates, and selected minerals) have been presented only for banana flour [41,99,101,102,104], some fruit and vegetables (Selecta orange, passion fruit, watermelon, lettuce, courgette, carrot, spinach, mint, taro, cucumber, rocket, and potato), residue flour [111,142], carrot puree [106], and ground cranberry pomace and pomace extract [112]. Different research was conducted by Reis, Souza, Silva, Martins, Nunes, and Druzian [129], who considered mango pulp as an antioxidant additive added at 10.0, 20.0 and 30.0%, for which more detailed chemical characterization was carried out (moisture, total solids, pH, total fiber, fatty acids, free sugars, total carotenoids, total polyphenols, and total flavonoids). Also, fruit and vegetable extracts are perceived as functional additives. They are usually added at various amounts: 0.5 g of total solids per 1 g of pectin (dry basis) for acerola, apple, and strawberry alcoholic extracts [83], 1.5, 3.0, 4.5, 6.0, and 10.0 g per 100 g total solution for apple skin polyphenol powder (apple skin extract) [87], and 4.0 and 8.0 mL per 100 mL total solution for grape pomace extract [116], 0.25, 0.50, 0.75, and 1.00 g per 100 g total solution for Indian gooseberry [120]. Subsequently, a chemical characterization is performed, assessing parameters such as total carotenoids, ascorbic acid, total phenolic compounds, and antioxidant capacity, either in line with anticipated functions [83] or, when dealing with purchased extracts, in line with the information that is readily available on the label [87,120]. It is very important to remember that such factors as crop species, variety, maturity, cultivation methods, and climate conditions exert a substantial influence on the chemical composition of fruits and vegetables, consequently affecting the properties of edible packaging [168]. A more comprehensive understanding of the precise composition of binding agents like pectins, cellulose, hemicellulose, lignin, starch, proteins, and plasticizers such as fructose, glucose, and sucrose, along with various functional compounds like antimicrobials, nutritional elements, and antioxidants in fruit and vegetable semi-products, would facilitate the improved design of edible materials. This knowledge would also enhance the ability to predict their properties, explain the outcomes of experiments, and enable meaningful comparisons between edible materials derived from different raw materials [169,170].

**Figure 6:**
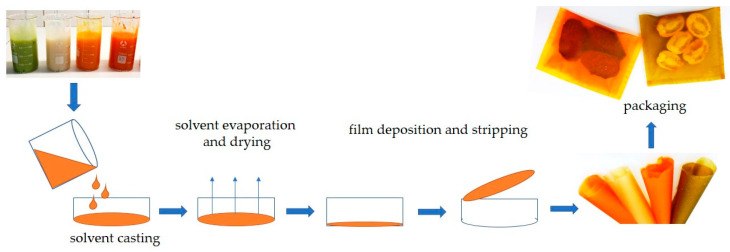
Casting method for producing edible films and sheets on a laboratory scale.

Otoni, Avena-Bustillos, Azeredo, Lorevice, Moura, Mattoso, and McHugh [74] noticed that the casting method (Figure 6), which is the most commonly used approach for producing edible films and sheets on a laboratory scale, becomes impractical when scaled up for industrial production due to excessively long drying times. They have suggested continuous casting as a promising technique for producing edible films and sheets, which allows the direct casting of the matrix-forming solutions onto a conveyor belt surface. Moreover, it can optimize uniformity, heat transfer, and drying efficiency, and significantly reduce drying times for this method of obtaining edible films. Another important issue is restricting the space needed for production to be more suitable for large-scale production. Du, Olsen, Avena-Bustillos, McHugh, Levin, and Friedman [146] produced tomato pectin films incorporated with carvacrol using these two different casting methods. They observed that continuous-cast films were thinner and denser than batch-cast films. Higher casting temperatures in the continuous method led to increased rates of carvacrol and water evaporation, likely resulting in reduced interstitial spaces within the matrix, and consequently lower water vapor permeability. Compared to batch casting, continuous casting had a positive impact on both tensile strength and elastic modulus values. Otoni, Avena-Bustillos, Azeredo, Lorevice, Moura, Mattoso, and McHugh [74] noted the scarcity of research on alternative techniques for producing edible films and sheets. This scarcity is primarily attributed to the thermosensitivity of the compounds in fruits/vegetables and biopolymers. Conversely, there have been scientific endeavors to optimize the production of fruit leather. Tontul and Topuz [137] conducted a study to assess the influence of various drying methods on the physicochemical properties of pomegranate leather. They aimed to address the limitations associated with traditional sun or shade drying, including extended drying times, the susceptibility to microbial contamination, and the reliance on environmental conditions. They investigated hot air drying at 50, 60, and 70 °C, microwave-assisted hot air drying at 50, 60, and 70 °C with power settings of 90 and 180 W, and reflectance window drying at 90, 95, and 98 °C. Among these methods, reflectance window drying emerged as the most favorable option, offering improved color and texture of the pestil, greater retention of nutritional and functional compounds, and reduced non-enzymatic browning reactions. What is more, the drying time shortened significantly from 135–250 min for hot air drying to 30–40 min for microwave-assisted hot air drying (180 W). The drying time for samples prepared using reflectance window drying was between 45 and 55 min. Concededly, drying times achieved using continuous drying were even as short as 12 min for tomato puree films [146]; however, more research is needed to optimize the conditions of edible film and sheet drying. The authors of this article would also like to indicate the necessity of detailed research regarding the technique and the parameters of matrix-forming formulation degassing, such as vacuum degassing [85], ultrasonic degassing [129], and resting [119], in terms of the viscosity of the formulation.

Nowadays, consumers’ demand for high food quality and its maintenance during the period between purchase and consumption is a very important issue for manufacturers. This necessity arises from the fundamental requirement of food safety, as well as from the need to minimize undesired changes in sensory attributes [171]. Fruits and vegetables are favored choices among consumers for their health benefits, yet they are living tissues and belong to the category of highly perishable goods, necessitating optimal post-harvest technologies to extend their shelf life and maintain storage stability. Numerous studies have reported low water activity values, much below 0.6 for edible materials derived from fruit and vegetable purees, rendering these products potentially microbiologically stable when stored under the appropriate dry conditions [47,127]. Consequently, incorporating fruits and vegetables as primary components in edible films and coatings offers the opportunity to enhance their stability while meeting consumer expectations [172]. On the contrary, despite extensive research on the shelf life and physicochemical properties of products coated or wrapped in edible fruit/vegetable materials, there is a notable gap in knowledge concerning the shelf life of edible films as standalone products. An exception is found in the work of Sothornvit and Rodsamran [127], who investigated the impact of mango films on the quality of whole and minimally processed mangoes. They reported no sugar crystallization in mango puree edible films stored at 30 °C for up to 3 months. Consequently, there is a pressing need for additional studies exploring the shelf life of edible films derived from fruits and vegetables; furthermore, this understanding should be informed by an understanding of the thermal and sorption characteristics of edible films, and so research is warranted to select suitable packaging materials and define optimal storage conditions (including relative humidity and temperature). This knowledge is crucial for the successful commercialization of such products. Last but not least, there is more and more research studying the possibility of the application of fruit and vegetable edible films/sheets as sealed food wraps [73,92,102,103,104,109]. The morphology of the surface has a great influence on the adhesion. Some authors have reported big differences in edible films, sheets, and leathers between the side facing upwards and the side facing downwards during drying [75,76,94]. The air-side surface was described as rough, while the basal side was smooth and shiny. Knowledge of the film morphology is very important while proposing possible applications of such structures such as, e.g., food wraps. A high level of surface roughness can diminish the degree of contact between the film and other materials, as well as within the film itself. This can result in the less effective sealing of the resulting structures. Edible films, which are dried for extended drying time, are characterized by a significant reduction in thickness due to water evaporation. When the rate of drying is quite low, the final products tend to be denser and more compact. Consequently, the molecules comprising the matrix of edible films are closely packed, potentially leading to reduced compressibility. This means that, even with adequate pressure and contact time, if the material is too rigid, the seal between two joined parts may be too weak, limiting the practical applications of edible films [94].

## 6. Sustainability–Advantages of Fruit- and Vegetable-Based Films, Sheets, and Coatings

Utilizing fruits and vegetables as key components in edible films, sheets, and coatings offers the advantage of repurposing raw materials with reduced commercial value, including deformed or slightly mechanically damaged produce, as well as smaller-than-standard items. This approach not only reduces waste, but also contributes to cost savings in the production process due to the lower cost of these fruits and vegetables [71]. Furthermore, during the production of fruit- and vegetable-based products, substantial quantities of residues, such as peels and seeds, must be efficiently processed to prevent the generation of easily decomposable waste [8,19,25,28,29,157]. Edible film, sheet, and coating formulations represent a novel approach to harnessing their potential [95]. The challenges posed by overproduction and the seasonal availability of certain fruits and vegetables can be effectively addressed by transforming fresh raw materials into edible films or sheets. These can serve as healthy snacks, offering microbiological stability for up to 6 months during storage, without the need for any preservatives, due to their intermediate water activity and low water content [58]. There is also the possibility of decreasing the amount of synthetic packaging waste using the application of fruit and vegetable films, sheets, and coatings simply as a passive or active layer, which could partially (with the current state of knowledge) replace nonrenewable materials [8,25,74,172]. Moreover, the utilization of fruits and vegetables in the form of puree or flour, without the necessity of the isolation of particular components, is also beneficial from a financial point of view [101]. Last but by no means least, there is an increasing demand for foodstuffs for particular nutritional uses, and in the opinion of the authors of this article, edible films and sheets have a great potential to serve as such products, for example, in the diet of people who are gluten-intolerant as an alternative to pancakes, tortillas, dumplings, or lavash dough.

## 7. Conclusions

In recent years, people have become more concerned about the sustainability and eco-friendliness of technologies. However, despite more than two decades having passed since the first publication about edible packaging utilizing various fruit purees, numerous uncertainties remain to be addressed, inquiries to be resolved, and challenges to be tackled to be able to introduce edible packaging onto the market. For this purpose, it is important to conduct a comprehensive analysis of the chemical composition of fruit and vegetable semi-products employed as constituents in matrix-forming solutions. Furthermore, some previously published research has already begun to delve into and elucidate the acquired data, citing the chemical composition of fruits and vegetables as a factor contributing to observed phenomena while emphasizing the need for further investigation in this area. Ongoing exploration into the characteristics of films derived from natural blends (flour, puree, and juice) and their comparison with films derived from components of the same botanical source promises to provide valuable insights into the nature of interactions occurring within the polymer matrix. Enhancing the understanding of edible films, sheets, and coatings derived from fruit and vegetable semi-products represents a promising avenue for scientific inquiry, aligning seamlessly with the principles of sustainable development.

## Figures and Tables

**Table 2:** Chemical composition of the selected fruits and vegetables [149,150,151,152,153].

Specification	Average Content [g/100 g FW]								
Fruits	Water	Total Protein	Fat	Total Carbohydrates	Total Sugars *	Starch	Dietary Fiber		
							Total	Soluble **	Insoluble ***
Apple	85.56	0.26	0.17	13.81	10.39	0.05	2.21	0.67	1.54
Strawberry	90.95	0.67	0.30	7.68	4.89	0.04	2.30	0.70	1.60
Sour cherry	86.13	1.00	0.30	12.18	8.49	0.00	1.50	0.60	0.90
Blackcurrant	81.96	1.40	0.41	15.38	9.00	0.00	7.90	^NA^	^NA^
Raspberry	85.75	1.20	0.65	11.94	4.42	0.00	5.50	2.88	2.62
**Vegetables**									
White cabbage	92.18	1.28	0.10	5.80	3.20	0.00	2.50	^NA^	^NA^
Carrot	88.29	0.93	0.24	9.58	4.74	1.43	2.88	0.49	2.39
Onion	89.11	1.10	0.10	9.34	4.24	0.00	1.93	0.71	1.22
Tomato	94.52	0.88	0.20	3.89	2.63	0.00	1.34	0.15	1.19
Red beet	87.58	1.61	0.17	9.56	6.76	0.10	2.80	^NA^	^NA^

^NA^–not available; * sum of saccharose, glucose, and fructose; ** sum of cellulose, hemicellulose, and lignin; *** sum of pectin, gum, and mucilages.

## Data Availability

Not applicable.
